# Approaching life in a test tube with prebiotic chemistry

**DOI:** 10.1038/s42004-026-02257-3

**Published:** 2026-10-23

**Authors:** Zbigniew L. Pianowski

**Affiliations:** 1 Institute of Organic Chemistry, Karlsruhe Institute of Technology KIT, 76131 Karlsruhe, Germany; 2 Institute of Biological and Chemical Systems—Functional Molecular Systems IBCS-FMS, Karlsruhe Institute of Technology KIT, 76131 Karlsruhe, Germany; 3 International Institute of Molecular Mechanisms and Machines IMol PAN, Polish Academy of Sciences, 02-247 Warszawa, Poland

**Keywords:** Origin of life, Self-assembly

## Abstract

The emergence of life on our planet occurred somewhere before 3.48 billion years ago, and was predated by a poorly defined period of appearance and accumulation of chemical intermediates commonly known as biomolecules – which includes amino acids, lipids, sugars and components of genetic polymers. While no fossils are known to science from that period of Earth’s development, prebiotic chemistry tried to experimentally answer the question: which chemical processes, and under which conditions, could provide our planet with biomolecules in sufficient quantity and structural diversity to enable spontaneous life formation. Here, we present a chronological overview of the hypotheses that have driven the development of prebiotic chemistry, and of seminal experiments which were intended to prove or disprove these hypotheses.

## Introduction

The phenomenon of life and its origins fascinated humans from the earliest times. The obvious difference between the animated and unanimated realms was initially explained by myths in each culture of our planet—ancient Egyptians, Mayas, Babylonian, Siberian, or Maori tribes - as well as by all organized religions. The big questions: “Where do we come from?”, “Are we alone in the Universe?”, or “Can we create life?” have inspired philosophers and artists. Yet, with the discovery that it is the DNA which turns dust into life, natural sciences found themselves in a position to tackle these questions based on the scientific methodology.

With that knowledge, we can tackle the question on how systems capable of storage and processing genetic information could evolve from random networks of chemical compounds with increasing complexity. This process on Earth took millions of years, under conditions which are still disputable - such as the early atmospheric composition, as well as pH, ion, or temperature gradients at specific geological setups. Yet, laboratory simulations performed within much shorter times, using limited amounts of purified starting materials, often demonstrate that experimental principles of simple molecular systems can be with high probability applied to much more complex setups. While many gaps between laboratory simulations and the early Earth environment still exist, prebiotic chemistry has already filled some of them with unexpectedly high accuracy, proving to be a promising approach for understanding the abiogenesis process.

While we are still far from giving the complete picture on how the unanimated matter could form first protocells on our planet, this review has an ambition to guide the readers through the most important experimental results which represent significant pieces of this large puzzle. While navigating through the particular stories, we have to remember that there is no direct way of verifying if one or the other experiment exactly repeats an event from the actual life formation period, as there are no known physical evidences, such as fossils, that could prove or disprove that. However, the experiments of today reflect certain “modes of actions” or patterns in the behaviour of the matter, which would be at least equivalent to the processes that had to occur on the way to primitive life.

An important philosophical consideration behind the research on the origins of life is the generality of this phenomenon. While the life on Earth is, by now, the only type of life known to the human kind, we consider it to be the result of a unique process. An alternative hypothesis is, that life – understood as the capacity of a molecular system to self-replicate and adapt to external conditions in an evolutionary way—might be emerging as a natural consequence of increasing complexity in any molecular system capable of information storage. If the latter is true, it would strongly indicate that the Universe is full of life forms (not necessarily intelligent, overwhelmingly microbial). It would also mean, that by acquiring sufficient understanding of the molecular principles behind that process one could create life de novo, in an experimental setup.

In this review, we aim to demonstrate—in a very compact form—the progress achieved until now in understanding the earliest molecular stages of the origins of life, up to the phase where it was ready to commence the Darwinian evolution. Even though this review is not intended as comprehensive, we hope that - in corroboration with more detailed sources^1–8^—the readers find the content of this manuscript inspiring for their future scientific research and experiments.

## The history of research into the origin of life

The first philosophical considerations on the nature of life and its origins are dated back to the times of classical Greek philosophers: the theory of spontaneous generation (abiogenesis), coined by Aristotle^9^ in his *History of Animals*, said that living creatures could arise from unanimated matter and that such processes were common and regular. Robert Hooke, the discoverer of a microscope, in 1665 published first drawings of bacteria. He viewed them as a proof of abiogenesis, as he believed them being spontaneously generated, and not multiplied by division. The theory of abiogenesis, although challenged in the 17th and 18th century by Italian biologists Francesco Redi and Lazzaro Spallanzani, was taken for a scientific fact until the experiments of French chemist Louis Pasteur^10^, who demonstrated that primitive organisms, such as bacteria and fungi, do not spontaneously appear in sterilized nutrient-rich media without inoculation from outside. He, with the support of John Tyndall, introduced the term “biogenesis” (life arises from existing life) as the opposition to the once-for-all disproven Aristotelian theory of spontaneous generation.

While it was clear that abiogenesis does not take place under experimental conditions today, many developmental biologists believed that a kind of spontaneous generation must have happened for the emergence of life on Earth. When Charles Darwin introduced his theory of natural selection in 1859, he did not explicitly discussed how life originated. Yet, in a letter to J.D.Hooker in 1871, Darwin mentioned a “warm little pond with all sort of ammonia and phosphoric salts,—light, heat, electricity present, where a protein compound was chemically formed, ready to undergo still more complex changes” as a potential place where life could appear for the first time on Earth^11^.

A coherent scientific argumentation to that picture has been introduced in 1924 by Alexander Oparin – a Soviet biochemist – who proposed a long process of chemical and pre-biological evolution, where the first forms of life should have been microorganisms dependent on the molecules and organic substances present in their external environment. According to him, on the surface of primitive Earth, the reducing atmosphere comprised of water vapour, ammonia, methane, and exposed to intense UV light and other energy sources could undergo reactions yielding first organic compounds, which could later assemble into proteins and other biomolecules. A particular type of colloid, called coacervate, would then appear by conglomeration of organic molecules and other polymers with positive and negative charges. Gradual evolution of the coacervates would ultimately yield the first organisms. This process was deemed impossible in the current oxygen-rich Earth atmosphere. Similar conclusions were independently drawn by an Indian-English scientist John B. S. Haldane in 1929^12^. The mature Oparin-Haldane hypothesis stated, that the chemically reducing Earth atmosphere exposed to energy (UV, lightnings) produced simple organic compounds (“monomers”), accumulated in a “prebiotic soup” (a term introduced by Haldane) which underwent concentration (shorelines, oceanic vents, “warm little pond”). And there more complex organic compounds (“polymers”) could form and eventually organize into the first protocell capable of Darwinian evolution^13^. The hypothesis was supported by experimental evidences first in mid-1900 by the Miller-Urey experiment (1952)^14^, and Joan Oró (1960) demonstrating abiotic synthesis of “monomers” - amino acids and adenine nucleobase^15^, respectively.

In 1909, Svante Arrhenius coined the name “panspermia”^16^ to the idea, originally presented by the Greek philosopher Anxagoras in 5th century BC, that life could arrive on Earth from the outer Universe. While this theory is merely speculative^17^, its modification (pseudo-panspermia) is the well-supported hypothesis saying that many of the small organic molecules (amino acids, nucleobases) needed for life’s emergence might be delivered to Earth by cosmic dust, asteroids, comets or planetoids impacting its surface^18–20^.

## The abiotic origins of biorelevant small molecules

Until the end of 20^th^ century, prebiotic chemistry was dominated by the reductionist approach, which assumed that each class of “monomers” building life has been generated merely independently from the others. And all they were only combined at the later stage into more complex setup, of which the genetic polymers and metabolism could emerge, leading finally to the first protocells. While this approach was initially promising, its inherent limitations became gradually obvious by the late 1980–1990s. The advent of 21st century complemented this approach by introducing the concept of systems chemistry^21^ which investigates networks of interacting molecules and their emergent properties^22,23^, also in the context of the origins of life^24^.

### Amino acids

Inspired by the Oparin-Haldane theory, Stanley Miller supervised by Harold Urey provided in 1952 the first experimental support to the abiotic generation of amino acids. The mixture of methane, ammonia, hydrogen in 2:2:1 ratio mixed with water vapour (simulating atmospheric composition of the early Earth) exposed to electric arc (simulating lightning) in a closed reaction vessel mimicking the hydrological cycle produced a complex mixture of compounds, including (racemic) amino acids (five in the original report)^14^. The early Earth atmosphere was at that time considered to be strongly reductive, which predetermined the choice of substrates used in the experiment. While oxidative atmosphere very likely appeared on Earth much after life already developed there (Great Oxygen Catastrophy caused by enormous evolutionary success of photosynthesizing cyanobacteria), the current consent opinion of geologists^25^ suggests that the early Earth atmosphere was more redox-neutral (based mainly on N_2_, CO, and H_2_O)^26,27^. Therefore, different experimental setups^28^ and less reductive composition of the gas mixture^29^ were tested. While generation of nitrites in CO_2_- and N_2_-rich atmosphere models was suspected to destroy amino acids, addition of buffering and reducing agents (CaCO_3_, ascorbic acid) significantly increased the overall amino acid yields^29^. Doping the mixture with H_2_S yielded total of 23 amino acids, including 6 sulfur-containing ones^30^.

**Fig. 1:**
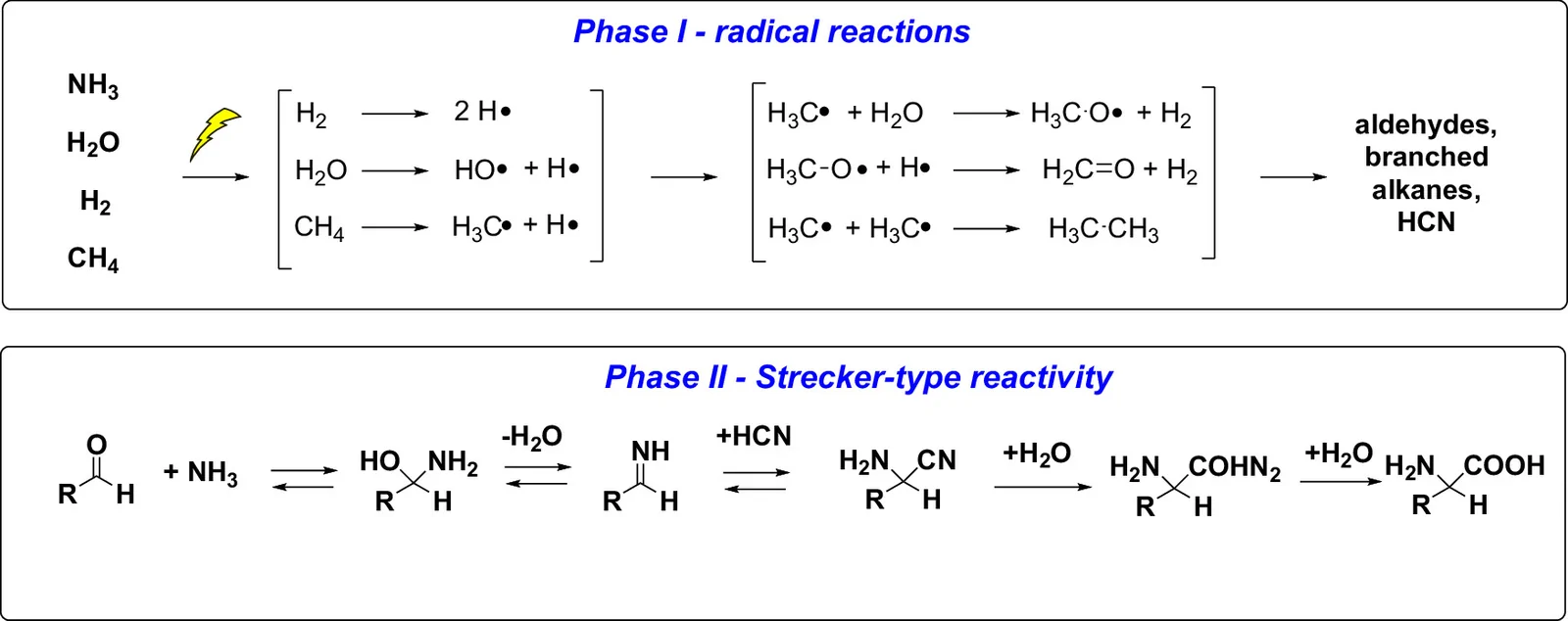
Miller-Urey experiment. Two main reactivity phases: the radical reactions resulting from homolysis of starting materials results in a mixture of recombination products, including aldehydes, branched alkanes, and hydrogen cyanide. In the following phase, aldehydes react with ammonia and hydrogen cyanide. The resulting α-aminonitriles undergo irreversible hydrolysis to α-amino acids. If HCN reacts directly with aldehydes via α-cyanohydrines, the following hydrolysis results in α-hydroxyacid products.

The Miller-Urey process has several distinct phases, where most important are: radical formation *via* UV light or electric discharge, their recombination into (often branched) aldehydes and HCN, and the Strecker reaction between these two intermediates in presence of ammonia, which ultimately generates the amino acids (Fig. 1).

Similar reactivity is likely responsible for the presence of amino acids detected in meteorites. The famous Murchison meteorite—a carbonaceous chondrite that fell in Australia in 1969, which particles are up to 7 billion years old—contains a large variety of C_2_-C_9_ amino acids, likely delivered i.a. by UV-light-catalyzed processes (producing carbonyl compounds) followed by Strecker reaction^31^.

Another plausible source of amino acids might be carbon fixation on catalytic transition metal precipitates under hydrothermal conditions – carbon monoxide on Ni(OH)_2_ under basic conditions in presence of cyanides produces a range of α-hydroxy and α-amino acids^32^.

While the aforementioned sources typically deliver racemic amino acid mixtures, the life on Earth uses predominantly the *L-*isomers to build proteins. The initial reasons for it remain obscure—whether very efficient amplification of stochastic imbalance^33^, or fundamental asymmetry of the Universe originating from the parity violation that provides one handedness with slightly lower energetic penalty than the other^34,35^. Much better understood is the following step—chirality amplification^36,37^. Already in 1953, Frederick Charles Frank postulated in his model, that homochirality is likely a consequence of autocatalysis^38^. The first experimentally demonstrated autocatalytic reaction leading to chirality amplification was an organometallic Soai reaction^39–41^. It was followed a decade later by reactions that can operate in aqueous systems, more relevant to the chemistry of life^42^, and complemented with pure physical phase separation processes—double crystallization of phenylalanine with 1% ee can deliver 90% ee product, profiting from the fact that the racemate is less soluble and can be merely separated as solid, while the initial minor ee imbalance in solution steadily increases^43^. Chirality amplification can be also mediated by chiral crystal surfaces of minerals (quartz, calcite), which can preferentially absorb one enantiomer of organic compounds, and thus affect the enantiomeric excess in supernatants, aggregates, polymers, or their relative degradation rate^44^. Interestingly, as chirality of organic molecules is coupled to their electron spins^45^, it was shown that ferromagnetic mineral surfaces can yield provide significant chirality amplification^46^.

Chiral amino acids and short peptides are very good enantioselective catalysts. They catalyse a range of asymmetric aldol, Mannich, Michael reactions, as well as hydrocyanations or even Strecker reactions themselves^47^—a concept broadly appraised today as organocatalysis^48^. Yet, at the advent of life on Earth, this feature might have facilitated propagation of the chiral imbalance into other classes of biorelevant molecules, such as sugars. Although it was demonstrated, that some rare cross-chiral systems can exhibit biological function^49^, general experimental evidences indicate that opposite enantiomers usually inhibit synthesis and/or proper function of homochiral biosystems^50^. Therefore, the current consent is, that efficient chirality amplification processes had to precede the emergence of complex functional biopolymers^35,51^.

Most likely, abiotic synthesis of short peptides occurred upon exposing amino acids on diverse dehydrating agents^52^. Volcanic gases, such as carbonyl sulfide (COS, or O = C = S), are the usual suspects, as they produce oligopeptides under mild conditions in aqueous solutions^53^. While some short peptides can self-assemble into β-sheet amyloid structures, which in turn may exhibit life-related functions, such as self-replication, catalysis, or information transfer, they are considered as potential entities that could stimulate the origin of life, in frame of the “amyloid world” concept^54,55^.

It has to be mentioned that the penultimate hydrolysis step of α-aminonitriles in the Strecker reaction is usually slow and often stops at the α-aminoamide stage^56^. The hydrolysis can be catalysed by carbonyl compounds (optimally formaldehyde)^57^. Alternative pathways were considered, including the Bucherer-Bergs reaction—forming hydantoine upon addition of CO_2_ and bicarbonate^58^, via N-carbamoyl-α-amino acids (NCA)^59^, or phosphoroaminonitriles (upon addition of diamidophosphate)^60^, yielding compounds that could be—in contrary to simple a-amino acids—energetically favourable intermediates in prebiotic peptide synthesis.

### Lipids

**Fig. 2:**
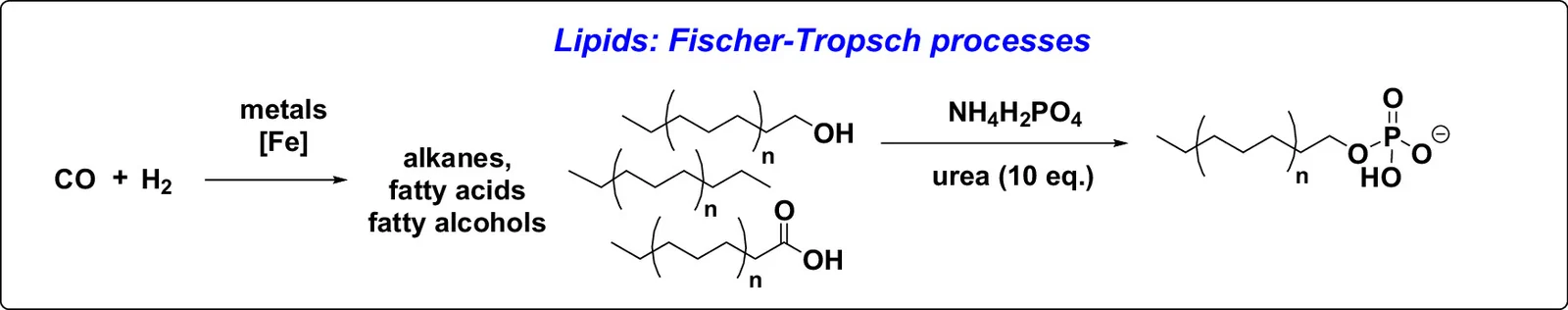
Prebiotic synthesis of lipids. Carbon monoxide and hydrogen condense on metal catalysts (such as meteoritic iron) to long alkanes, and lipids (fatty acids, fatty alcohols). These can be further functionalized, using e.g., prebiotic phosphorylation conditions.

Evolving chemical systems requires compartments capable of Darwinian evolution—to compete, to store information, and to concentrate metabolites. The compartments can be provided by lipids—amphiphilic organic compounds which tend to spontaneously form vesicles in water. Among many lipids known to biology, fatty acids and fatty alcohols are the most likely to occur prebiotically and be used to form first protocells. The Fischer-Tropsch-type synthesis (reaction of CO and H_2_ in presence of transition metal catalysts) is generally acknowledged to be the considerable, of not the main source of prebiotic lipids^61^. While industrially its conditions are directed towards hydrocarbons, n-fatty acids and long-chain alcohols can be already formed by passing syngas over meteoritic iron. Fischer-Tropsch-type processes can also occur under hydrothermal conditions at moderate temperatures (150–250 °C)^62^. Upon addition of ammonia to the mixtures, various amino acids are also detected in the reaction mixtures^63^. Phosphates accessible upon corrosion of meteorites^64,65^ can also phosphorylate alcohols, preferentially long-chain ones, in presence of urea, yielding primitive phospholipids (Fig. 2)^66^. A variety of lipids has been also detected in meteorites^67,68^, so that vesicles form even upon simple mixing of meteorite extracts with water^69^.

Experiments show, that even simple fatty acids can emulate basic cellular behaviour quite accurately, as uncomplicated lipid vesicles can already grow and divide spontaneously (upon mechanical stimulation, or by photoredox processes)^70–72^. In contrary to the diacyl phospholipids building modern cells, vesicles made of monoacyl lipids are permeable to polar molecules^73,74^. This likely enabled selective uptake of various protocell components in the absence of complex protein channels^75^. In light of potential protocell formation in the mineral-rich Archean Ocean, it was important to show that while some lipid vesicles with primitive composition retain their stability in presence of divalent cations (like Mg^2+^) at high concentrations (up to 10–50 mM)^76^, others can be stabilized upon addition of prebiotically relevant amino acids^77^. Diacyl phospholipids seem to additionally enhance the vesicle stability towards flocculation and drying^78^. Short amphiphilic oligopeptides can efficiently attract oligonucleotides to the lumen of lipid vesicles, which makes a plausible location for chemical evolution of ribozymes and the advent of RNA world^79^. Moreover, it was demonstrated that blending various types of lipids can provide vesicles stable towards divalent cations, sufficiently permeable for small charged molecules such as nucleotides, and compatible with nucleic acid synthesis^80^ and stability (like nonenzymatic RNA replication^76,81^ or ribozyme catalysis^82^). External stimuli such as UV light or shaking^71,83^, as well as internal chemical reactions^72,84^ can influence growth and fission of simple lipid vesicles, while freezing-thawing cycles enabled material exchange between diverse vesicle populations^85^—arts of behaviour that are typical for living cells.

Finally, while lipids certainly encapsulated early unicellular organisms on Earth, some hypotheses state that they were not forming the compartments that witnessed the first sparks of life. Based on the fact that bacterial and archaeal membranes significantly differ from each other by composition (fatty acids vs. isoprenoids), it was suggested that the first life might have evolved in immobile compartments, such as porous rocks of hydrothermal vents, fuelled with temperature and ion gradient. And only later, synthesis of lipid membranes (which occurred independently for bacterial and archaeal ancestors) enabled detachment of the organisms from the solid support and their autonomous existence in aqueous milieu^86^.

### Sugars

**Fig. 3:**
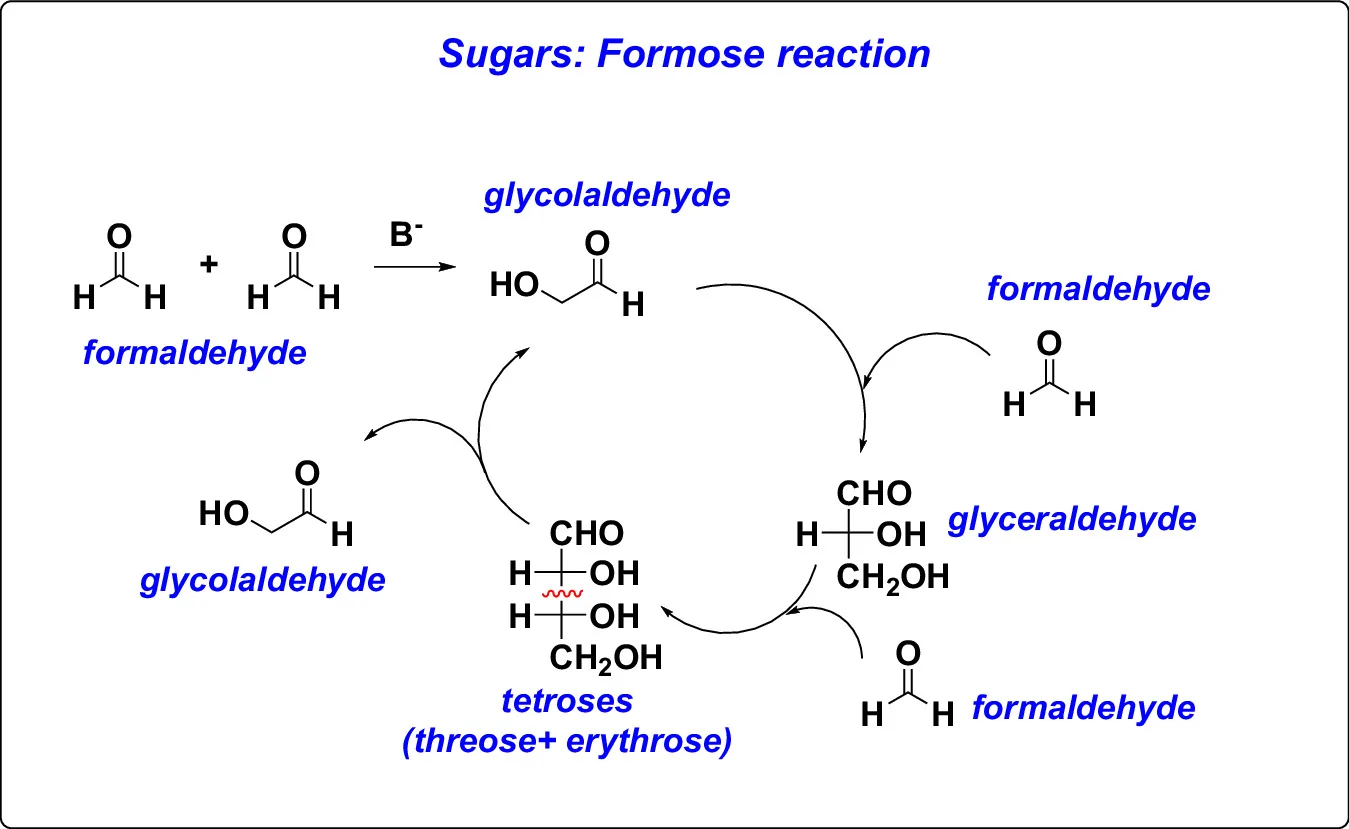
Formose reaction—an autocatalytic cycle proposed by R. Breslow in 1959. The base-catalysed condensation of formaldehyde to aldoses and ketoses, discovered in 1861 by A. Butlerov, was the first process considered to be a prebiotic source of ribose and other simple sugars. The initially slow self-condensation of formaldehyde yields glycolaldehyde, which reacts faster with following formaldehyde molecules via aldol-type condensations and rearrangements, yielding C_3_, C_4_, and higher sugars. Tetroses undergo i.a. retro-aldol reaction which produces two glycolaldehyde molecules and renders the reaction autocatalytic.

Sugars and their derivatives are important components of current metabolic cycles, as well as genetic polymers—RNA and DNA. Historically, the first process suspected of delivering sugar building blocks for life was formose reaction (Fig. 3)—already in 1861, Alexander Butlerov demonstrated^87^ that heating formaldehyde under basic conditions with divalent metal cations (Ca, Mg) results in a mixture of sugars, including tetroses, pentoses, and hexoses. In 1959, Ronald Breslow proposed that this reaction is autocatalytic and explored details of its mechanism^88^. Yet, it usually produces a large diversity of products, and – if not stopped early enough – insoluble polymers. Generation of numerous compounds might support certain stages in the assembly of proto-metabolism^89^, but would rather be an obstacle for prebiotic formation of RNA. The mechanism of formose reaction is still under vivid debate, and its meaning for prebiotic chemistry spans beyond simple sugar formation^90–92^. Recently, it was demonstrated that some minerals (especially borates) can actually stabilize the formose reaction outcome towards ribose and other pentoses^93^, but thermodynamic and kinetic obstacles of various nature make it unlikely to deliver components of first genetic polymers on Earth^94^.

Ribose and related sugars were also observed in experimental simulations of interstellar ice formation comprising water, methanol, and ammonia, exposed on UV irradiation^95^.

The availability of ribose-stabilizing minerals, such as borates, under prebiotic conditions is disputable^96^—they occur in felsic rocks of continental crust, rather than in mantle and oceanic crust, yet some were found in 3.8-billion-years-old metasediments in Grenland^97^. Yet, the prebiotic applicability of formose reaction as the ribose source has other critical issues – very difficult first step of formaldehyde dimerization, general lack of control over product mixtures^98^, and - at best—moderate stability of borate-ribose complexes under the reaction conditions^99,100^.

**Fig. 4:**
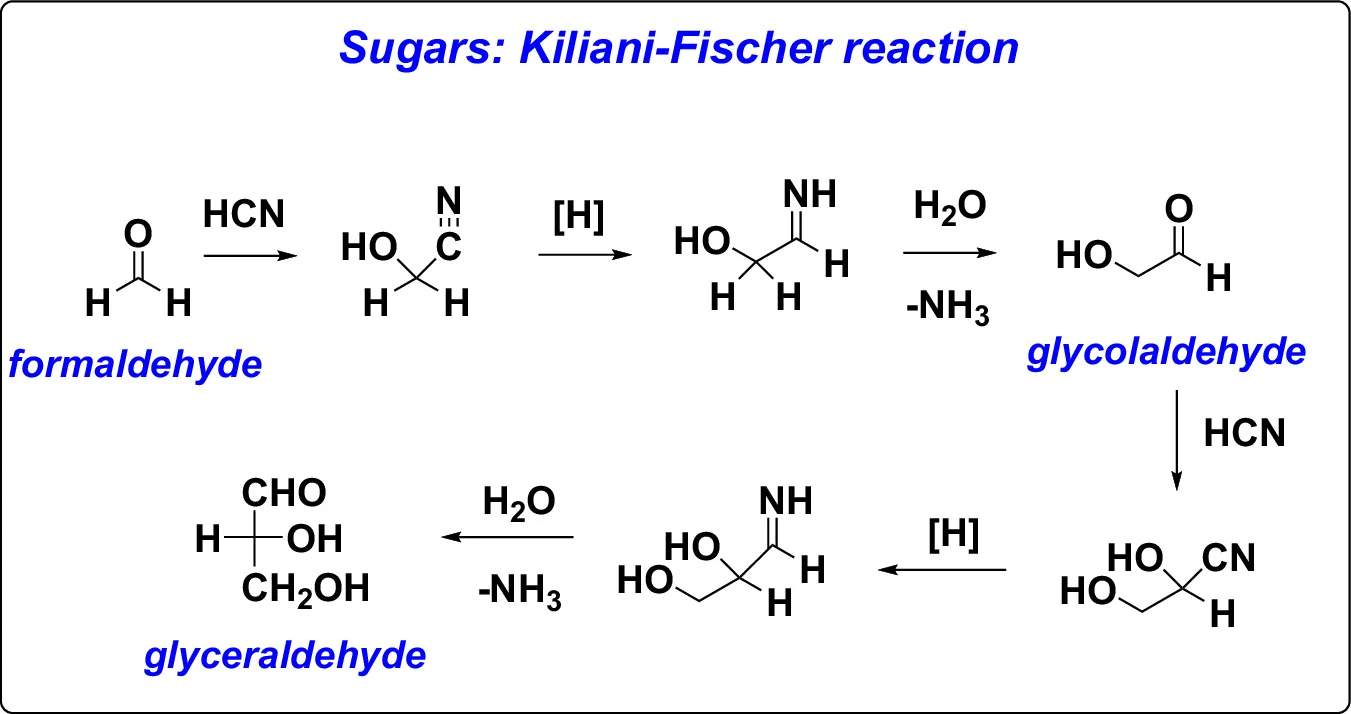
Kiliani–Fischer reaction. Formaldehyde (accessible also via reduction of HCN) reacts quickly with HCN. The resulting cyanohydrin can be reduced to the α-hydroxyimine which hydrolyses to glycolaldehyde. The latter can react with additional HCN molecule and produce (via reduction and hydrolysis) glyceraldehyde. Under prebiotic conditions it was proposed that the reduction can be performed by cyanocuprate complexes in a UV-light-catalyzed photoredox process.

Recently, it was suggested that well-defined sugar precursors of RNA can originate from Kiliani-Fischer type of reactivity, starting from C_1_ sources such as formaldehyde or HCN that undergo photoredox-induced homologation (catalysed by cyanocuprate complexes) to glycolaldehyde and glyceraldehyde, which react further to provide the pentose derivatives (Fig. 4)^101,102^. The advantages of Kiliani–Fischer reaction in comparison to formose reaction comprise favourable kinetics of the C_2_-intermediate (cyanohydrin) formation, lack of formaldehyde disproportionation with the related material losses, and stepwise addition of following C_1_-fragments in form of HCN, alternated with photoredox-driven generation of the next aldehyde homologue. It can also occur under mild (pH 7, phosphate-buffered) condition, which minimizes product degradation^101,103^.

### Nucleobases and nucleosides

**Fig. 5:**
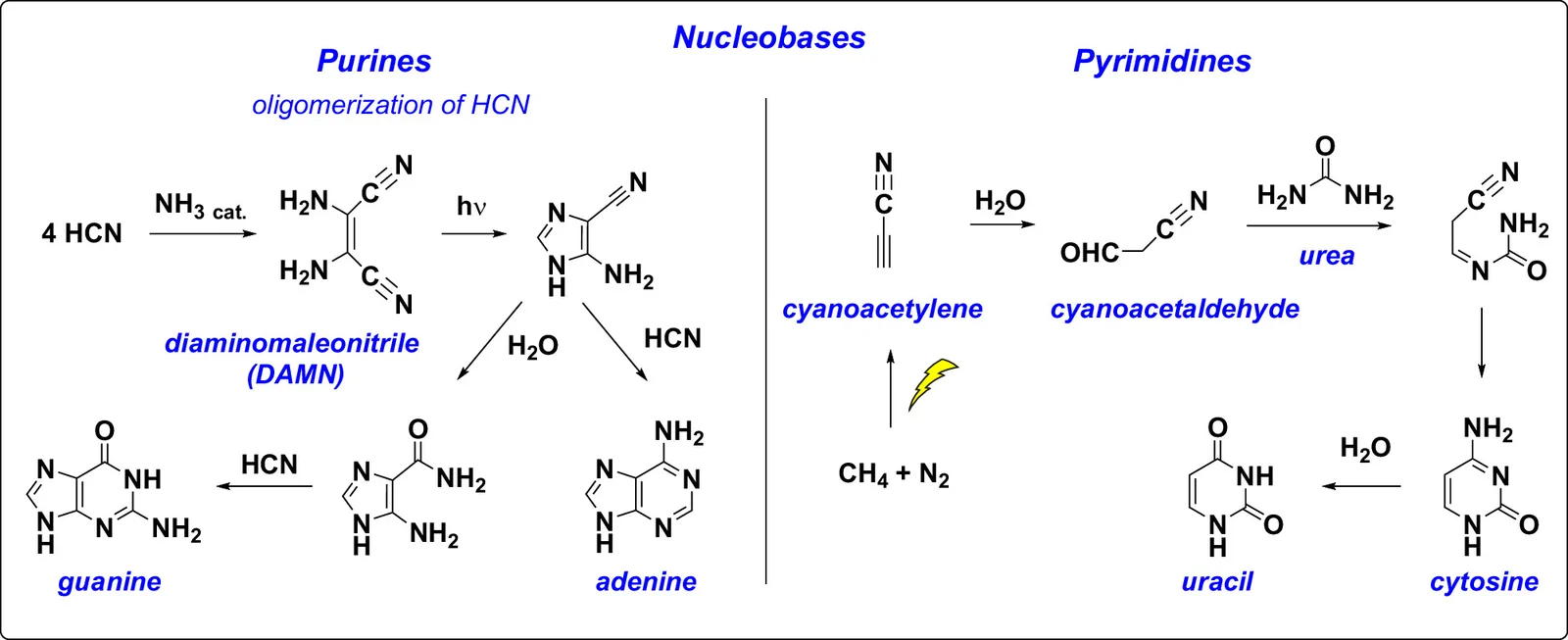
Prebiotic synthesis of nucleobases. Purines can be obtained from ammonia-catalysed oligomerization of HCN, while pyrimidines result from cyanoacetylene (product of electric discharge in a mixture of nitrogen and methane), which—after partial hydrolysis—can react with urea to form cytosine. Cytosine can hydrolyze to uracil.

Prebiotic synthesis of nucleobases—the second component of nucleosides—has been shown as early as 1960s. Joan Oro demonstrated^15,104^ that adenine can spontaneously form by oligomerization of hydrogen cyanide catalysed by aqueous ammonia. Other purines, including guanine, appear as side products^105^. Pyrimidines follow a distinct synthesis pathway - cytosine is formed e.g., by the reaction of cyanoacetylene with urea^106^, and uracil is the hydrolysis product of cytosine (Fig. 5).

**Fig. 6:**
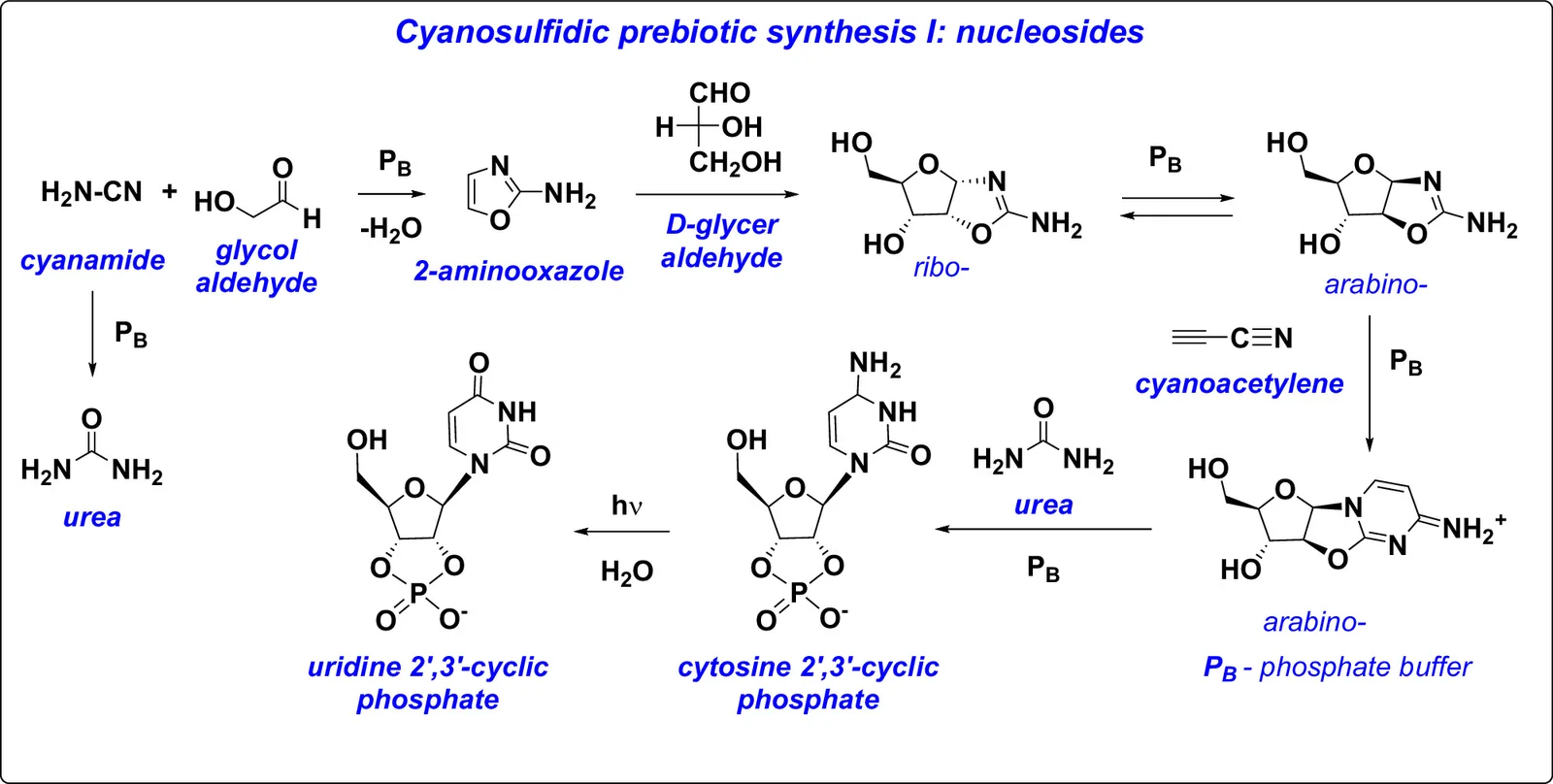
Cyanosulfidic prebiotic synthesis of pyrimidine nucleosides. Stepwise condensation of glycolaldehyde and glycerylaldehyde (derived e.g., from Kiliani–Fischer reaction) with cyanamide, when buffered with phosphate, yields partially protected aldopendoses, which upon reaction with cyanoacetylene and urea produce pyrimidine nucleosides activated as 2’,3’ cyclic phosphates. Urea – the side product of the first condensation – is a substrate for the later step.

**Fig. 7:**
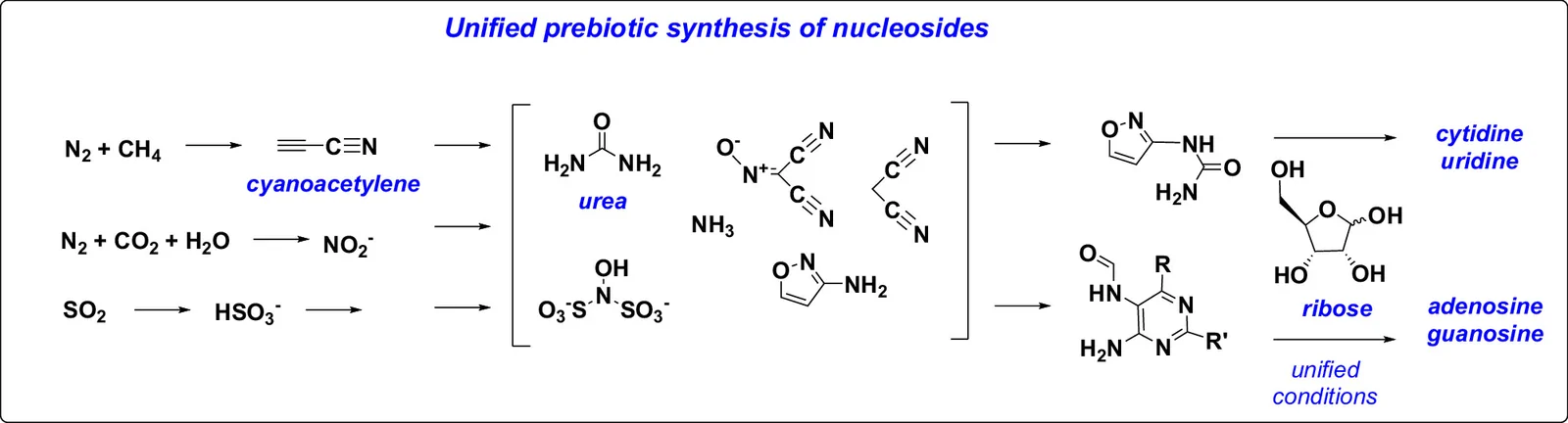
Unified prebiotic synthesis of nucleosides. Formamidopyrimidines in presence of ribose undergo condensation into purine nucleosides. Pyrimidine nucleosides can be obtained under the same conditions from ribose and heterocyclic isoxazole precursors. These intermediates can be plausibly obtained from simple gases of partially volcanic origin.

While plausible synthetic routes to nucleobases have been demonstrated, for a long time the community struggled with the problem of combining them with sugars into canonical ribonucleoside structures. No efficient reaction conditions have been found for that until now. Instead, brilliant concepts have been provided and experimentally proven, where nucleosides are constructed stepwise from nucleobase precursors around sugars. One concept—cyanosulfidic chemistry (Fig. 6)—proposed by John D. Sutherland, provided an impressively clean process to pyrimidine nucleosides from several reactive intermediates, including glycolaldehyde, glyceraldehyde (both traced back to HCN), and cyanoacetylene in phosphate buffer, catalysed with urea^107^, also in an enantioselective fashion^108^. An alternative system from Thomas Carell relied initially on the condensation of formamidopyrimidines with ribose^109^, which was later complemented with hydroxylamine-based chemistry which allowed for unified conditions yielding all four canonical nucleosides via wet-dry cycles (Fig. 7)^110^.

Yet, the demonstrated reaction pathways strongly depend on precise regulation and sequential additions of reactants, which might be difficult to achieve under non-laboratory prebiotic conditions. For cyanosulfidic chemistry, it was shown that addition of 2-aminothiazole can significantly improve selectivity of the desired products from the complex mixtures of substrates *via* transient aminal formation, additionally providing a novel synthetic pathway to proteinogenic amino acids^111^.

### Systems chemistry—cyanosulfidic prebiotic synthesis

**Fig. 8:**
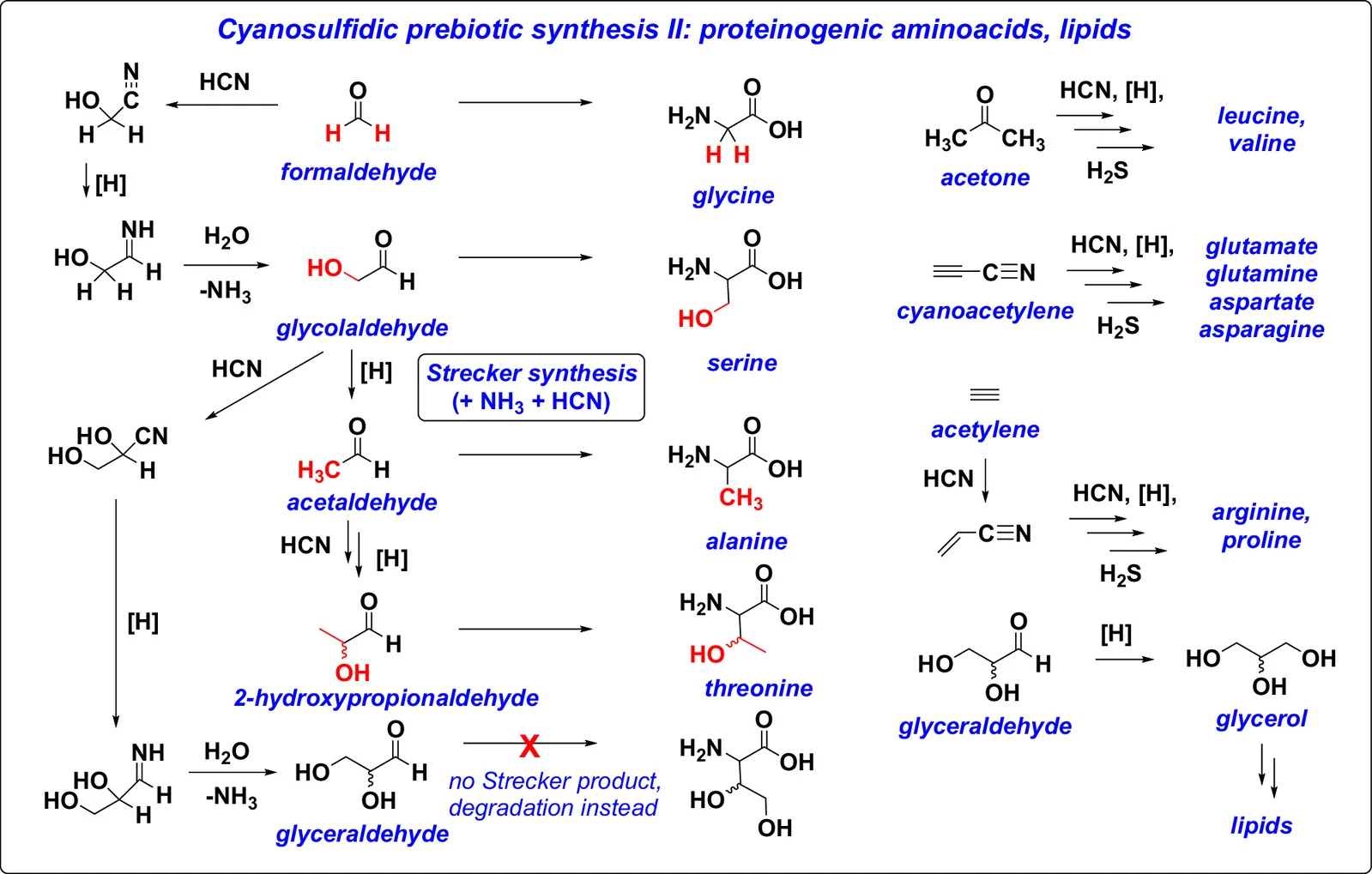
Cyanosulfidic prebiotic synthesis of proteinogenic amino acids and lipids. Overreduction of nucleoside precursors yields acetaldehyde, 2-hydroxypropionaldehyde, acetone and glycerol. All the aldehydes can be converted to proteinogenic amino acids via Strecker reaction (yet glyceraldehyde – which would yield a non-proteinogenic 3-hydroxythreonine – undergoes degradation under Strecker conditions). Photoredox processes in presence of HCN yield further proteinogenic amino acids and lipid precursors via glycerine and its cyclic phosphates.

The concept of systems chemistry^21^ which analyses networks of interacting molecules and their emergent properties which can lead to new functions absent in individual components^22,23^, has been introduced in contradiction to the reductionist analysis of prebiotic chemistry. While typical approach of organic chemistry assumes working on small number of pure substrates, often highly concentrated, and perform a unitary process with high yields of a single or main product, it poorly reflects the most likely realms of earliest life formation phases. It is almost certain, that life emerged from complex mixtures of multiple components fuelled by potent sources of energy (chemical, electrical, or light), likely as a result of sequential chemical reactions, or dynamic steady states that provided constant influx of labile intermediates. The first complex system of mutually reactive intermediates originating mainly from hydrogen cyanide, which provides a broad range of biologically relevant “monomers” simply by diverging the components and catalysts present in the environment, has been reported by the group of John D. Sutherland. There, in presence of metal complexes, UV light, simple molecules (phosphates, urea), and reducing agents related with volcanic gases (including H_2_S), hydrogen cyanide can be converted into a range of proteinogenic amino acids, lipid components^112^, and pyrimidine nucleosides – elementary processes take place under relatively mild conditions in presence of water and provide good yields of clean products, if performed in the correct order^113^. This work was ground-breaking for prebiotic chemistry, as it practically demonstrated that components of various biopolymers can be synthesized from common precursors, and thus might have emerged in parallel and not from less probable coincidence of several independent processes (Fig. 8).

In the aforementioned examples of chemical systems, the authors typically propose that the order of reagent addition should be determined by the geological conditions (often depicted as streams of water sequentially dissolving reagents from geological deposits such as evaporated minerals) combined with day/night cycles as a way of oscillatory delivery of UV light into photoredox catalytic systems, or evaporation/resolubilization of certain components or mixtures. These graphically appealing representations unveil the complexity and vulnerability of systems chemistry approach and modular synthetic pathways to the chosen environmental conditions. Larger input is needed especially from deep analysis of the kinetics of unitary reactions involved in such systems to be considered prebiotically plausible^114,115^. Another important aspect is the compatibility and stability of products from different synthetic pathways, as well as the likelihood of their accumulation in a shared environment^116–118^. That, in turns, relates to the long-standing debate if life emerged spontaneously from different levels of organization in a prebiotic soup of components^119^, or *via* free energy feeding provided by geological gradients^120^—the issue discussed below in more details.

## Prebiotic sources of biopolymers

**Fig. 9:**
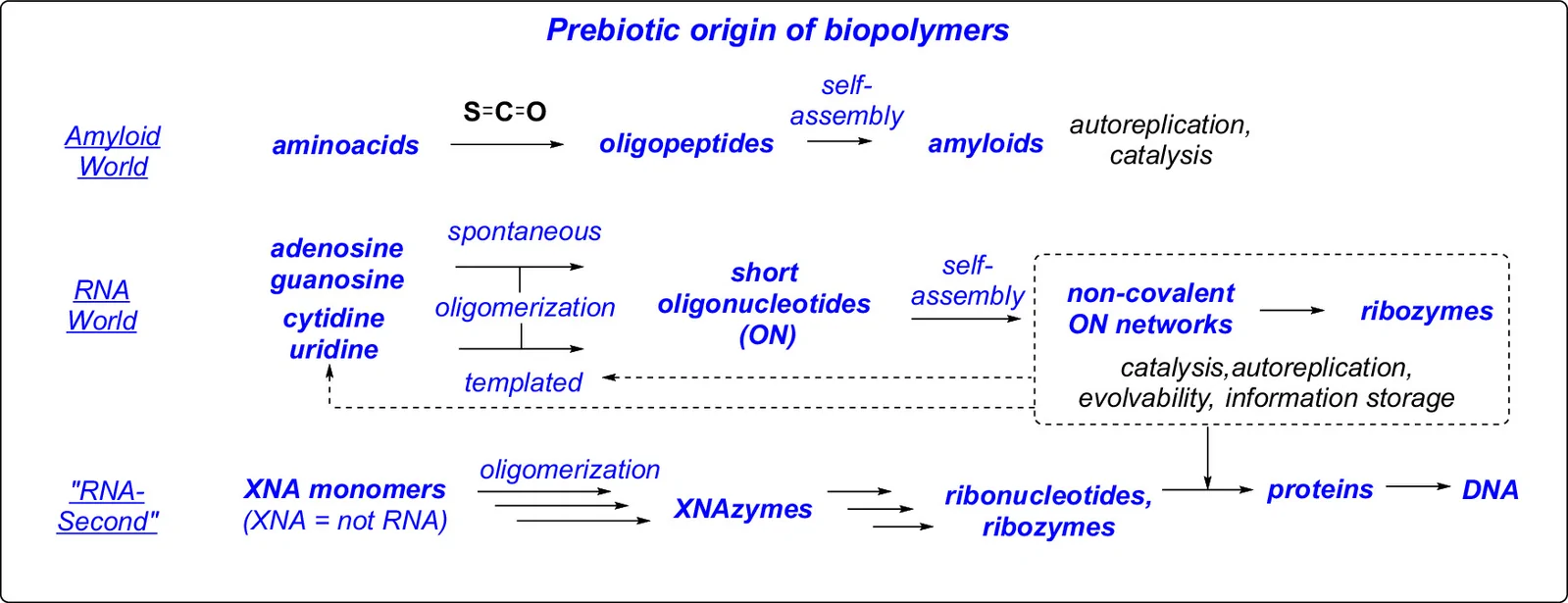
Prebiotic origins of biopolymers. Oligopeptides lack a general mechanism of autoreplication, yet this ability can occur for specific supramolecular aggregates – amyloids – capable of catalysis, adaptability to environmental changes, and molecular information storage. Nucleosides (vide supra) might have spontaneously oligomerize, and the products assemble into non-covalent oligomeric networks, in which specific functions (catalysis, (auto)replication capacity) slowly emerged. The non-covalent aggregates possibly turned into specific covalent ribozymes. Both stages could template the synthesis of more ribonucleosides and RNA oligomers, forming autocatalytic systems. Other (simpler, more robust) genetic polymers (such as peptide nucleic acids PNA) might pre-date RNA in assembling autocatalytic evolvable systems.

While monomeric amino acids and nucleosides possess some catalytic activity and well-defined chemical reactivity, the complex functions such as selective catalysis or information storage can only emerge with well-defined long polymers (enzymes, ribozymes, genetic material). In contrary to the prebiotic generation of biorelevant “monomers”, the process of sequence-specific abiotic polymerization is less-well understood. While condensation of amino acids yielding random oligopeptides can be catalysed with volcanic gases^53^, abiotic sequence-specific formation or proteins or long peptides is much less probable. In particular, lack of general mechanisms for templated protein replication may indicate that proteins are relatively late addition to life’s evolution. In specific cases, however, one can identify peptides capable of self-replication—based on coiled coil motif^121^ or amyloids^54^. These observations led to formulation of “Amyloid world” hypothesis (C.P.J. Maury, 2009)^122,123^, where the first functional polymers on the primitive Earth were structurally stable self-replicating beta-sheet peptide structures^124^. Amyloids based on short peptides demonstrated structural stability, (auto)catalytic properties, evolvability, enantioselectivity, and error correction capability (Fig. 9)^125,126^. One of the challenges in the “Amyloid world” concept is the pre-requisite for spontaneous accumulation of multiple sets of identical (or very similar) peptides at high concentrations, in order to form a diverse collection of functional amyloids with mutually collaborative functions.

An alternative hypothesis is the idea of “RNA world”^127^ which states that RNA – due to its capacity for genetic information storage, and the ability for 3D-folding into diverse conformations with catalytic propensities - was the first polymer of life that enabled evolution of protocells, prior to recruiting proteins for enhancing its catalytic abilities (Fig. 9). That hypothesis^128^, originally a speculation of Carl Woese, Francis Crick^129^ and Leslie Orgel^130^ from 1960’s, gained momentum with the observation of Harold B. White III^131^ that ribonucleotide co-enzymes present in active centres of many enzymes may be “living fossils” from the primordial RNA-based metabolism originally performed without proteins. Another evidence came from the discovery of ribozymes^132^ – RNA enzymes - by Thomas Cech^133^ and Sidney Altman^134,135^, (Nobel prize in 1989). The strongest proof for that theory came with the advent of the new millennium^136,137^. In 2009 Ada Yonath, Venkatraman Ramakrishnan, and Thomas A. Steitz shared a Nobel prize for studies of the structure and functions of ribosome. Using high-resolution crystal structures, they demonstrated that ribosome is a ribozyme, and thus that it is RNA which “learned” how to synthesize proteins, and not the opposite.

While we are still far from producing the first protocell in the lab, certain stages of that process have been already experimentally investigated, and compose into an increasingly comprehensive picture. The single nucleosides, which might have emerged from processes described in the previous sections, could undergo oligomerization. The biological process, where the sequence and regioselectivity of polymerization is strictly controlled by the template and polymerase enzyme, respectively, could not occur in the prebiotic manner with a similar level of pre-organization. Yet, certain degree of control can be achieved already under simple reaction conditions. James Ferris showed that certain clays (montmorillonite) can provide decent regiocontrol of nucleotide oligomerization due to preorganization within its crystal structure lattice^138^. Autocatalytic amplification of oligonucleotides (although limited to short oligomers due to required level of pre-organization) has been demonstrated by Günter von Kiedrowski^139^. Sequence control in the absence of polymerases can be achieved in the primer extension format, explored by Jack Szostak^140^. Sutherland lab not only demonstrated that it is possible to direct the regioselectivity of template RNA oligomer ligation using acetylating reagents^141^, but also that the “wrong” 2’-5’ junctions can be “repaired” by a non-enzymatic equilibration process to the physiologically relevant 3’-5’ phosphodiester bonds^142^. There, the selectivity comes from preferential 2’-O-acetylation of 3’-monophosphate-terminated oligomers in comparison to their 2’-photophorylated regioisomers, coupled with favourable hydrolysis rates of the “incorrect” 2’,5’-phosphodiester bonds and other unwanted byproducts – altogether concerted in cycles of acetylation, ligation, and hydrolysis in carbonate-buffered aqueous solutions with 40 mM Mg^2+^ ions at ambient temperature. The “proofreading” of 2’,5’-linkages could enable abiotic reshuffling and recombination of short RNA fragments, thus enhancing the emergence of functional RNA oligomers. By modification of solvent viscosity, the product inhibition can be supressed in the case of longer oligomeric template replication, as demonstrated by Nicolas Hud^143^.

Altogether, these observations give us already a hint that the abiotic assembly of longer oligonucleotides with correct regioselectivity might occur spontaneously under relatively simple reaction conditions. However, there is still a large gap in understanding on how such transition could occur. The later stages of this process have been recently unveiled by investigation of engineered ribozymes. While their naturally occurring counterparts can currently achieve simple ligations or cleavage of sequence-defined RNA strands, artificially generated ribozymes can efficiently catalyse template-directed replication of RNA templates via primer extension (either using 2’-3’-cyclic phosphates of nucleosides^144^, or nucleoside triphosphates) with gradually increasing length of the product: from 14-mer in 2001^145^ to the oligomers exceeding in length the ribozyme itself (>207 nt) in 2013^146^ – as demonstrated by groups of David Bartel and Philipp Holliger. Yet, it is unclear how such long catalytic RNA replicases (RNA-templated RNA polymerase ribozymes) with complex 3D fold could spontaneously emerge from simple (activated) nucleosides. Currently, the most plausible hypothesis suggests the intermediate stage of autocatalytic networks of non-covalent aggregates of medium-size (20–50-nt) spontaneously generated RNA oligomers. Due to their partial self-complementarity, the sufficiently large “library” of such middle-size fragments could probably spontaneously generate minor amounts of aggregates capable of particular enzymatic/replicatory/self-replicating activities. If this outcome is coupled with a yet unidentified amplification mechanism, these aggregates over time could evolve into covalently linked long (>200 nt) fully functional ribozymes, including the replicases and a proto-ribozyme. This hypothesis is supported by experiments on analysis and artificial assembly of RNA-based autocatalytic networks^147^, as reported by Gerald Joyce or Niles Lehman. In particular, the *Azoarcus* recombinase ribozyme can be assembled from four 43-65-nt-long RNA strands using short complementary internal guided sequences (IGS)^148^, which prompted research of engineering even more complex RNA networks^149^. *Azoarcus*-derived catalytic RNA networks were also demonstrated in coacervates^150^, which would be even more relevant to the evolution of first protocells. Summarizing, the likely way from unitary (activated) nucleosides to full-size RNA ribozymes capable of fine catalysis and self-replication is initial spontaneous regioselective oligomerization followed by assembly of increasingly complex non-covalent (auto)catalytic networks^151^ from middle-size RNA oligomers, which was concluded with covalent binding of the fragments into distinct functional entities^152^. Yet, it has to be noted that this hypothesis, although attractive, remains highly speculative – the main challenges here would be identification of “meaningful” RNA aggregates and their subsequent stabilization and amplification, along with aforementioned general hydrolytic lability of RNA oligomers. One suggested solution here is compartmentalization of such RNA networks in coacervates^150^, possibly in presence of short peptides and metal ions^153^.

Another serious problem of the “RNA world” hypotheis is the emergence of both stable and meaningful RNA under the presumably very harsh environment of the early Earth. Due to limited ex vivo stability of RNA mono- and oligomers, it was also proposed that RNA world might have been pre-dated by “XNA world”^154^ based on more stable and robust genetic polymers (xeno nucleic acids, XNA^155^), which evolved in severe environment, and only later has been replaced by RNA-based systems/protocells (Fig. 9). The question, why Nature finally relied on RNA and DNA as canonical genetic polymers out of multiple combinations of nucleobases, sugars and their linkers, was theoretically and experimentally addressed shortly after the discovery of DNA and RNA structures. This endeavour, which resulted in numerous functional (that is, capable of storing and processing genetic information) oligonucleotide analogues, has been elegantly reviewed by Albert Eschenmoser^156^. XNAs generally retain canonical nucleobases, while the ribose-phosphate backbone is modified—slightly, as in ANA (arabinose instead of ribose) or FANA (fluoroarabinose), more drastically (ribose replaced by threose or cyclohexene in TNA or CENA), or very significantly—like in PNA (peptide nucleic acid), or GNA (glycol nucleic acid) where the whole backbone is replaced by a peptoid^157^, or by 1,2-propanediol^158^, respectively. Some types of XNA (HNA, CeNA, LNA, TNA) can be recognized by mutated polymerase enzymes and mutually transcribed into or from DNA complements^159^, they and other XNAs (like FANA, or ANA) can form receptors^160^, aptamers and XNAzymes, which not only act on specific RNA sequences, but can also catalyse reactions on other XNA strands^161^. XNAs bearing tetrose, or alternative pentose backbone could, in principle, follow prebiotic synthetic pathways already explored in the context of ribose, although e.g., no mineral-driven stabilization of formose products other than ribose have been reported up till now. Yet, evidences for prebiotic emergence of PNA or GNA, and their existence under “harsher conditions” in Nature, are at best scarce. PNA backbone (2-aminoethyl-glycine) has been detected as one of the products in Miller-Urey-type experimental setup^162^, and as a metabolite of cyanobacteria, but without specific proof of its involvement in the genetic apparatus^163^. If RNA is the evolutionary offspring of simpler XNA genetic polymers, this transition could have been driven by factors such as increased catalytic capacity of RNA, more efficient biosynthesis using (proto)metabolic processes, or better adaptability to environmental changes, while already protected by well-developed protocells from premature degradation.

While it is unclear, also in light of recently reported prebiotically plausible syntheses of nucleotides mentioned above^107,110^, if XNAs (particularly the robust ones such as PNA or GNA) are direct evolutionary ancestors of RNA, their discovery and investigation seriously contributed to our understanding of genetic polymers in general, but also provided numerous useful probes for biology and medicine (FISH probes, artificial antisense oligonucleotides) that can interact with native oligonucleotides and remain stable towards enzymatic degradation^164,165^.

The contradiction of peptide- and nucleotide-based systems seems to recently loose its momentum, as numerous researchers have identified systems, where interplay between these two realms, as well as nucleopeptides and lipid structures overcomes the bottlenecks of chemical evolution previously identified for systems composed only or peptides or only of nucleotides. In such co-evolutionary pathways, each component mutually enhances the efficiency of self-assembly, replication, catalysis, or energy dissipation^166–171^.

## Prebiotic origins of metabolism

The core structure of metabolism is very similar across all known organism, which suggest its early evolutionary origin^172^. The question of prebiotic formation of (proto)metabolism has been initially considered somewhat separately from the origins of genetic materials described above. General considerations on spontaneous emergence of reaction networks capable of converting chemical evolution into protobiology pertained to the autocatalytic formose-like processes, or steady-state systems propelled by geological energy sources (volcanic gases, hydrothermal vents, minerals). The discussion: “metabolism-first^173^ vs. genetics-first” was vivid in the 20th century—the earlier supported by Morowitz^174^ or Wächtershäuser^175^, the latter – by Orgel. One of the debated issues was, if nucleotides could emerge and form the RNA world in a spontaneous process, or would this event require previous formation of a protometabolism capable of constant delivery of chemical energy and basic RNA building blocks. That useful discussion was somehow sharpened by the arguments pointing at the presumed complexity of functional RNA molecules (supposedly impossible to assemble without existing protometabolism) and the lack of evolutionary pressure strong enough to assemble a complete metabolic network before life came into existence via genetics^176,177^. Due to enormously complex experimental setups required for (dis)proving either of these arguments, the discussion was – out of necessity—heavily speculative. Yet, the last two decades brought a number of important experimental evidences that partially unveiled the routes by which protometabolism could have emerged. Currently, the consent opinion is, that relatively complex chemical reaction networks can spontaneously form in a steady state under abiotic conditions, and slowly dissipate chemical energy via several sugars or carboxylic acids. Once spontaneously generated, these chemical networks could have been taken over by emerging protocells and directly used for gradual rather than rapid chemical energy dissipation (catabolism), possibly fuelling other simple chemical cycles which might enhance the production of genetic polymers. The next step is supposedly increased control over the chemical networks using isolated enzymes (initially ribozymes), which finally assembled into fully matured metabolic pathways that we know from primitive living organisms nowadays. Importantly for the emergence and development of protometabolism in the RNA world context, recent results indicate that RNA-derived coenzymes, such as pyridoxal phosphate, adenosine diphosphate, and nicotinamide adenine dinucleotide, can mediate key metabolic reactions in the absence of enzymes^178–181^.

**Fig. 10:**
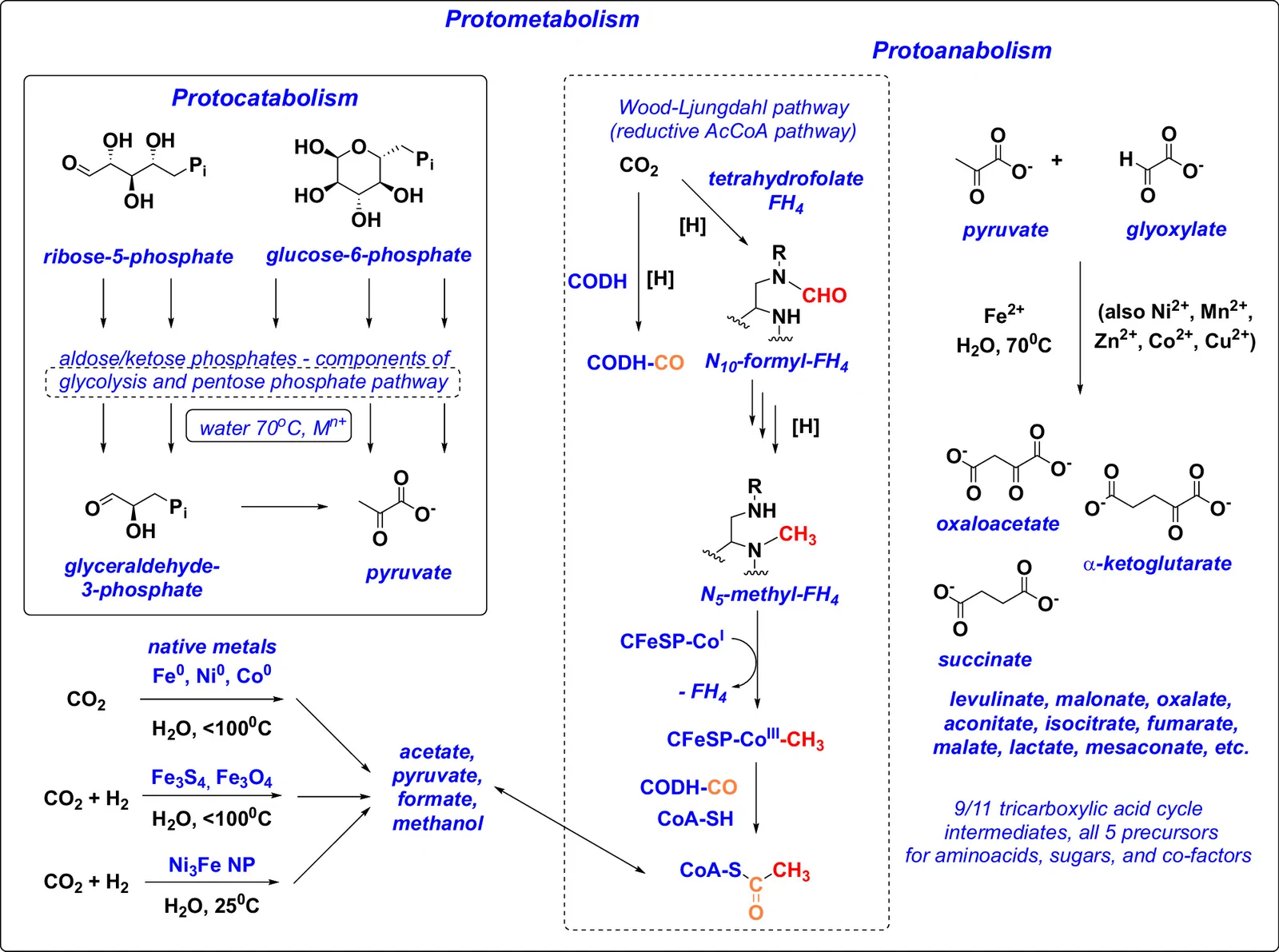
Prebiotic origins of metabolism. Currently existing metabolic pathways might have likely evolved from spontaneous reactions occurring under abiotic conditions in aqueous media in presence of simple transition metal catalysts. Stepwise degradation of sugar phosphates in warm water resembles glycolysis and other catabolic pathways, while CO_2_ in water on native metals or minerals yields numerous C_2_ and C_3_ intermediates (which resembles the current reductive AcCoA pathway’s chemistry). These, in turn, may enter a chemical system (under aqueous conditions, with metal ions) that – in the steady state – produces a majority of tricarboxylic acid cycle intermediates. These compounds may be likely abiotic precursors to numerous amino acids, sugars, or enzymatic co-factors, under conditions that were partially demonstrated experimentally.

The other dimension was added to the discussion with demonstrating spontaneously emerging chemical networks capable of inorganic carbon fixation. Here as well, abiotic chemical reactivity of CO_2_ was converted into a stepwise system comprised of enzymatically catalysed processes, ultimately combined into a coherent metabolic pathway (Fig. 10).

First experiments which processed along this line of thinking focused on abiotic formation of simple C_1_-C_2_ intermediates present as elements of currently known metabolic pathways (such as thioacetates as analogues of acetyl-coenzyme A - AcCoA) from inorganic sources, possibly under hydrothermal conditions or fuelled with ion gradients^182^. More complex examples of biorelated chemical networks, spontaneously generated under abiotic conditions, were demonstrated in 2014. The group of Markus Ralser showed^183,184^ a network of non-enzymatic glycolysis and pentose phosphate pathway-like reactions which could emerge in hot water in presence of simple mineral catalysts, which composition was compared to an Archeal ocean. Shortly afterwards, in 2017 Joseph Moran lab demonstrated that numerous intermediates present in the tricarboxylic acid cycle do not only spontaneously arise, but can also interconvert under non-enzymatic conditions using simple metal ion catalysis^185^. This report was followed by an expanded work showing metal-promoted (iron, nickel, meteoritic metals) synthesis and breakdown of universal metabolic precursors in absence of enzymes^186^. There, a reaction of glycolate and glyoxalate generates a steady state composed of over a dozen of metabolically related compounds under abiotic conditions^187^. This work was complemented with demonstration of non-enzymatic amino acid synthesis from ketoacids^188^, and work of Krishnamurthy and Springsteen groups^189^ on prebiotic metal-free analogues of the Krebs cycle^189^.

About the same time, William F. Martin recapitulated the role of carbon-metal bonds in the most ancient metabolic pathways, which corroborates with the hypotheses on the emergence of protometabolism using simple mineral catalysts^190^, and was supported by phylogenetic analysis^191^. This work was corroborated by the Moran group demonstrating that geochemical CO_2_ fixation on native transition metals (Fe, Ni, Co) could have supplied critical C_2_ and C_2_ metabolites (acetate, pyruvate) in water (40–100 °C, 1-40 bar CO_2_) before the emergence of enzymes^192^. A mixture of CO_2_ and H_2_ (available *via* serpentinization) also produces acetate and pyruvate in presence of hydrothermal minerals^193^, or on Ni_3_Fe nanoparticles – the latter even at room temperature^194^.

An increasingly popular concept of hydrothermal vents as the geological environment of life emergence is very appealing, as these energy- and mineral-rich formations provide gradients of temperature, pH, and metal ions that could drive the formation of protometabolic chemical networks based on simple organic compounds compartmentalized e.g., inside porous minerals^191^. The protometabolism could initially operate with native transition metals (or minerals^195^) and/or simple nucleotide-based cofactors *in lieu* of enzymes^196^. Over time, more protocell components could gradually accumulate. The energy in form of pH gradients between alkaline vent water and more acidic seawater could be at certain moment used, along with catalytic iron nickel sulfide minerals, to reduce CO_2_ and produce organic molecules with increasing level of complexity, up to self-replicating and catalytic molecules. At later stages, the pH gradient could be harnessed by enzymes (protein-based, and beforehand maybe of ribozyme nature) analogous to ATP synthases, to produce intermediates rich in chemical energy (such as ATP or its functional analogues), which were in turn capable of propelling the remaining parts of protometabolic cycles. Such process would mirror chemiosmotic coupling – the major pathway of ATP synthesis in extant organisms^197^. The following evolution of ion pumps (primitive proton pumps, or a sodium/proton antiporter) could decrease the dependence of such systems from external proton gradients, enable less permeable (e.g., phospholipid) membranes, and enable its transition from mineral compartments into free-living protocells suspended in water^86,198^. Interestingly, this hypothesis also offers an explanation for diverse membrane composition of bacteria and archaea, with retained core internal biochemistry^86^. In the ongoing discussion, the likelihood of escape of such organisms from natural pH gradient environment such as alkaline hydrothermal vents is also critically analysed^199,200^.

## Conclusions

In the previous paragraphs, we provided an overview on the recent achievements and disputes in the area of prebiotic chemistry. While the fascinating considerations on the interplay of early biopolymers resulting in the formation of first protocells, or the possible features of LUCA (Last Universal Common Ancestor) were not demonstrated here due to space limitations, they could only become possible at the moment, when a firm influx of biopolymers coming from abiotic sources has occured, and the ability for self-replication, information storage, and adaptability to the changing environment emerged from initially static collections of chemical compounds. While the phenomenon of life is still unique, the longer we investigate the propensity of small molecules for organization into well-defined supramolecular structures, the more probable is the hypothesis that life might be simply an emergent property of the matter that assembles into sufficiently complex chemical systems.

## Data Availability

The manuscript contains no original data. All data discussed in this manuscript can be found in the respective references.
